# Supplementary material for: Snail augments fatty acid oxidation by suppression of mitochondrial ACC2 during cancer progression
Source: Life Sci Alliance. 2020 Jun 2;3(7):e202000683. doi: 10.26508/lsa.202000683 (PMC7283136; doi:10.26508/lsa.202000683)
Supplement: Supplementary file 3 [file LSA-2020-00683_Supplemental_Data_2.docx]

**Supplementary data links**

**Snail augments fatty acid oxidation by suppression of mitochondrial ACC2 promoting breast cancer progression**

| **BRCA** | [**http://gdac.broadinstitute.org/runs/stddata__2016_01_28/data/BRCA/20160128/gdac.broadinstitute.org_BRCA.Merge_rnaseqv2__illuminahiseq_rnaseqv2__unc_edu__Level_3__RSEM_genes_normalized__data.Level_3.2016012800.0.0.tar.gz**](http://gdac.broadinstitute.org/runs/stddata__2016_01_28/data/BRCA/20160128/gdac.broadinstitute.org_BRCA.Merge_rnaseqv2__illuminahiseq_rnaseqv2__unc_edu__Level_3__RSEM_genes_normalized__data.Level_3.2016012800.0.0.tar.gz) |
| --- | --- |
| **COADEAD** | [**http://gdac.broadinstitute.org/runs/stddata__2016_01_28/data/COADREAD/20160128/gdac.broadinstitute.org_COADREAD.Merge_rnaseqv2__illuminahiseq_rnaseqv2__unc_edu__Level_3__RSEM_genes_normalized__data.Level_3.2016012800.0.0.tar.gz**](http://gdac.broadinstitute.org/runs/stddata__2016_01_28/data/COADREAD/20160128/gdac.broadinstitute.org_COADREAD.Merge_rnaseqv2__illuminahiseq_rnaseqv2__unc_edu__Level_3__RSEM_genes_normalized__data.Level_3.2016012800.0.0.tar.gz) |
| **HNSC** | [**http://gdac.broadinstitute.org/runs/stddata__2016_01_28/data/HNSC/20160128/gdac.broadinstitute.org_HNSC.Merge_rnaseqv2__illuminahiseq_rnaseqv2__unc_edu__Level_3__RSEM_genes_normalized__data.Level_3.2016012800.0.0.tar.gz**](http://gdac.broadinstitute.org/runs/stddata__2016_01_28/data/HNSC/20160128/gdac.broadinstitute.org_HNSC.Merge_rnaseqv2__illuminahiseq_rnaseqv2__unc_edu__Level_3__RSEM_genes_normalized__data.Level_3.2016012800.0.0.tar.gz) |
| **KIPAN** | [**http://gdac.broadinstitute.org/runs/stddata__2016_01_28/data/KIPAN/20160128/gdac.broadinstitute.org_KIPAN.Merge_rnaseqv2__illuminahiseq_rnaseqv2__unc_edu__Level_3__RSEM_genes_normalized__data.Level_3.2016012800.0.0.tar.gz**](http://gdac.broadinstitute.org/runs/stddata__2016_01_28/data/KIPAN/20160128/gdac.broadinstitute.org_KIPAN.Merge_rnaseqv2__illuminahiseq_rnaseqv2__unc_edu__Level_3__RSEM_genes_normalized__data.Level_3.2016012800.0.0.tar.gz) |
| **LIHC** | [**http://gdac.broadinstitute.org/runs/stddata__2016_01_28/data/LIHC/20160128/gdac.broadinstitute.org_LIHC.Merge_rnaseqv2__illuminahiseq_rnaseqv2__unc_edu__Level_3__RSEM_genes_normalized__data.Level_3.2016012800.0.0.tar.gz**](http://gdac.broadinstitute.org/runs/stddata__2016_01_28/data/LIHC/20160128/gdac.broadinstitute.org_LIHC.Merge_rnaseqv2__illuminahiseq_rnaseqv2__unc_edu__Level_3__RSEM_genes_normalized__data.Level_3.2016012800.0.0.tar.gz) |
| **LUAD** | [**http://gdac.broadinstitute.org/runs/stddata__2016_01_28/data/LUAD/20160128/gdac.broadinstitute.org_LUAD.Merge_rnaseqv2__illuminahiseq_rnaseqv2__unc_edu__Level_3__RSEM_genes_normalized__data.Level_3.2016012800.0.0.tar.gz**](http://gdac.broadinstitute.org/runs/stddata__2016_01_28/data/LUAD/20160128/gdac.broadinstitute.org_LUAD.Merge_rnaseqv2__illuminahiseq_rnaseqv2__unc_edu__Level_3__RSEM_genes_normalized__data.Level_3.2016012800.0.0.tar.gz) |
| **LUSC** | [**http://gdac.broadinstitute.org/runs/stddata__2016_01_28/data/LUSC/20160128/gdac.broadinstitute.org_LUSC.Merge_rnaseqv2__illuminahiseq_rnaseqv2__unc_edu__Level_3__RSEM_genes_normalized__data.Level_3.2016012800.0.0.tar.gz**](http://gdac.broadinstitute.org/runs/stddata__2016_01_28/data/LUSC/20160128/gdac.broadinstitute.org_LUSC.Merge_rnaseqv2__illuminahiseq_rnaseqv2__unc_edu__Level_3__RSEM_genes_normalized__data.Level_3.2016012800.0.0.tar.gz) |
| **PRAD** | [**http://gdac.broadinstitute.org/runs/stddata__2016_01_28/data/PRAD/20160128/gdac.broadinstitute.org_PRAD.Merge_rnaseqv2__illuminahiseq_rnaseqv2__unc_edu__Level_3__RSEM_genes_normalized__data.Level_3.2016012800.0.0.tar.gz**](http://gdac.broadinstitute.org/runs/stddata__2016_01_28/data/PRAD/20160128/gdac.broadinstitute.org_PRAD.Merge_rnaseqv2__illuminahiseq_rnaseqv2__unc_edu__Level_3__RSEM_genes_normalized__data.Level_3.2016012800.0.0.tar.gz) |
| **STAD** | [**http://gdac.broadinstitute.org/runs/stddata__2016_01_28/data/STAD/20160128/gdac.broadinstitute.org_STAD.Merge_rnaseqv2__illuminahiseq_rnaseqv2__unc_edu__Level_3__RSEM_genes_normalized__data.Level_3.2016012800.0.0.tar.gz**](http://gdac.broadinstitute.org/runs/stddata__2016_01_28/data/STAD/20160128/gdac.broadinstitute.org_STAD.Merge_rnaseqv2__illuminahiseq_rnaseqv2__unc_edu__Level_3__RSEM_genes_normalized__data.Level_3.2016012800.0.0.tar.gz) |
| **THCA** | [**http://gdac.broadinstitute.org/runs/stddata__2016_01_28/data/THCA/20160128/gdac.broadinstitute.org_THCA.Merge_rnaseqv2__illuminahiseq_rnaseqv2__unc_edu__Level_3__RSEM_genes_normalized__data.Level_3.2016012800.0.0.tar.gz**](http://gdac.broadinstitute.org/runs/stddata__2016_01_28/data/THCA/20160128/gdac.broadinstitute.org_THCA.Merge_rnaseqv2__illuminahiseq_rnaseqv2__unc_edu__Level_3__RSEM_genes_normalized__data.Level_3.2016012800.0.0.tar.gz) |

Ji Hye Yang^1,4,5^, Nam Hee Kim^1,4^, Yoon Mi Lee^1^, Eunae Sandra Cho^1^, Yong Hoon Cha^2^, Jun Sup Yun^1^, Hee Eun Kang^1^, Sue Bean Cho^1^, Seon-Hyeong Lee^3^, So Young Cha^1^, Soo-Youl Kim^3^, Jiwon Choi^1^, Hyun Sil Kim^1,*^ & Jong In Yook^1,*^
